# Draft Genome Sequence of the Aquatic Fungus *Margaritispora aquatica* Strain NNIBRFG339

**DOI:** 10.1128/MRA.00866-19

**Published:** 2019-11-07

**Authors:** Jaeduk Goh, Hye Yeon Mun, Young-Hwan Park, Sangkyu Park, Yoosun Oh, Namil Chung

**Affiliations:** a Fungi Research Team, Nakdonggang National Institute of Biological Resources, Sangju, Republic of Korea; Broad Institute

## Abstract

Margaritispora aquatica is an aquatic fungal species found in leaf litter. Here, we report the 42.5-Mb draft genome sequence of M. aquatica strain NNIBRFG339, which comprises 61 scaffolds and has an overall G+C content of 45.77% and an *N*_50_ value of 1.856 Mb.

## ANNOUNCEMENT

Margaritispora aquatica (family Incertae sedis, order Helotiales) is a common aquatic fungus and is one of the lignolytic fungi associated with decomposition of leaf litter in streams (1, 2). M. aquatica strain NNIBRFG339 was originally isolated from plant litter in Youngju, Republic of Korea (3). Here, we report the first draft genome sequence of this useful freshwater fungus.

A mycelial plug grown on potato dextrose agar (PDA; BD, Franklin Lakes, NJ, USA) at 20°C was used as the initial inoculum for the liquid culture. Mycelia were then grown in potato dextrose broth in the dark at 20°C with continuous agitation at 180 rpm. Genomic DNA and total RNA were extracted from the mycelia using a DNeasy minikit (Qiagen, Valencia, CA, USA) and the easy-spin total RNA extraction kit (iNtRON, Sungnam, Republic of Korea), respectively. The genome sequence of strain NNIBRFG339 was obtained through a combination of four PacBio RS II single-molecule real-time (SMRT) cells (total of 684,558 reads and 3,578,834,968 bp), one paired-end library (total of 23,200,650 reads and 2,343,265,650 bp), and one mate pair library (total of 34,155,925 reads and 3,449,748,425 bp) on a NovaSeq 6000 Illumina platform (DNA Link, Seoul, Republic of Korea). The PacBio library and a paired-end Illumina library with an insert size of 350 bp were constructed after DNA fragmentation, A tailing, phosphorylation, and adapter ligation. A mate pair Illumina library with an insert size of 20 kb was constructed with the Nextera mate pair library preparation kit (Illumina, San Diego, CA). A transcriptome sequencing (RNAseq) library was constructed with random hexamer priming by paired-end sequencing after fragmentation. Read quality was checked using FastQC (http://www.bioinformatics.babraham.ac.uk/projects/fastqc). Raw PacBio reads were preprocessed using Hierarchical Genome Assembly Process (HGAP.3) v.2.3.0 with genome size and minimum seed length parameters of 35 Mb and 9 kb, respectively (4). Paired-end and mate pair reads were preprocessed using bcl2fastq2 v.2.20. Draft genome sequences were assembled with HGAP.3 v.2.3.0 using PacBio data (default parameters), and error correction with whole-genome sequencing (WGS) paired-end reads was performed by using the FastaAlternateReferenceMaker tool for generating an alternative reference sequence over the specified interval after the application of GATK (v.4.0) UnifiedGenotyper and variantEval for variant evaluation (default parameters) (5). Scaffolding of the Nextera mate pair library was performed with NextClip v.1.3.1 (6) (parameters, -n = 600,000,000; -m = 25; -t = 19; -x = 34, 18; y = 32, 17) and SSPACE v3.0 (7) (parameter set, -x = 1; -m = 30). RNAseq reads were mapped to the assembled genome using TopHat v.2.1.1 (8).

The draft genome of NNIBRFG339 comprised 61 contigs with an *N*_50_ value of 1.856 Mb and a coverage depth of 65.66× (calculated using PacBio reads). The total length of the assembled genome was 42,517,347 bp, with a G+C content of 45.77%. The maximum and minimum scaffold lengths were 3,350,688 and 1,332 bp, respectively. This draft genome sequence will support functional genetic research on the life cycle of lignolytic fungi.

### Data availability.

The draft genome sequence of *M. aquatica* strain NNIBRFG339 has been deposited in GenBank under accession no. VJWN00000000. The SRA accession numbers are SRR9668956, SRR9669368, SRR9669410, and SRR9695842. This paper describes the first version of the genome for this strain.

## References

[1] Ingold CT 1942 Aquatic hyphomycetes of decaying alder leaves. Trans Br Mycol Soc 25:339–417. doi:10.1016/S0007-1536(42)80001-7.

[2] Abdel-Raheem AM, Ali EH 2004 Lignocellulolytic enzyme production by aquatic hyphomycetes species isolated from the Nile’s delta region. Mycopathologia 157:277–286. doi:10.1023/b:myco.0000024178.62244.7c. 15180156

[3] Mun HY, Goh J, Oh Y, Chung N 2016 New records of three aquatic fungi isolated from freshwater in Samcheok and Yeongju, Korea. Kor J Mycol 44:247–251.

[4] Chin CS, Alexander DH, Marks P, Klammer AA, Drake J, Heiner C, Clum A, Copeland A, Huddleston J, Eichler EE, Turner SW, Korlach J 2013 Nonhybrid, finished microbial genome assemblies from long-read SMRT sequencing data. Nat Methods 10:563. doi:10.1038/nmeth.2474. 23644548

[5] Li H 2014 Toward better understanding of artifacts in variant calling from high-coverage samples. Bioinformatics 30:2843–2851. doi:10.1093/bioinformatics/btu356. 24974202 PMC4271055

[6] Leggett RM, Clavijo BJ, Clissold L, Clark MD, Caccamo M 2014 NextClip: an analysis and read preparation tool for Nextera long mate pair libraries. Bioinformatics 30:566–568. doi:10.1093/bioinformatics/btt702. 24297520 PMC3928519

[7] Boetzer M, Henkel CV, Jansen HJ, Butler D, Pirovano W 2011 Scaffolding pre-assembled contigs using SSPACE. Bioinformatics 27:578–579. doi:10.1093/bioinformatics/btq683. 21149342

[8] Kim D, Pertea G, Trapnell C, Pimentel H, Kelley R, Salzberg SL 2013 TopHat2: accurate alignment of transcriptomes in the presence of insertions, deletions and gene fusions. Genome Biol 14:R36. doi:10.1186/gb-2013-14-4-r36. 23618408 PMC4053844

